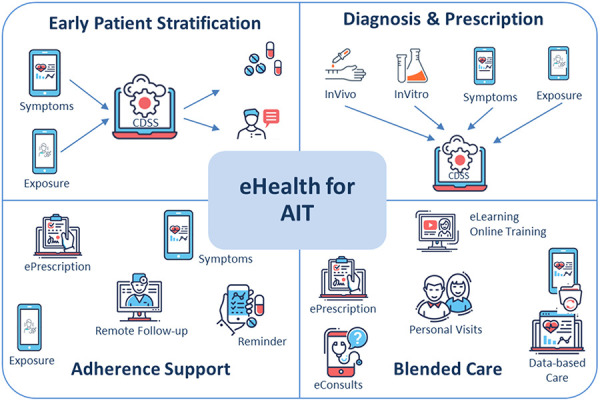# Digital health for allergen immunotherapy 

**DOI:** 10.5414/ALX02301E

**Published:** 2022-12-05

**Authors:** Stephanie Dramburg, Paolo Maria Matricardi, Oliver Pfaar, Ludger Klimek

**Affiliations:** 1 Department of Pediatric Respiratory Medicine, Immunology and Critical Care Medicine, Charité – Universitätsmedizin Berlin, Berlin,; 2 Department of Otorhinolaryngology, Head and Neck Surgery, Section of Rhinology and Allergy, University Hospital Marburg, Philipps-Universität Marburg, Marburg, and; 3 Center for Rhinology and Allergology Wiesbaden, Wiesbaden, Germany

**Keywords:** allergen immunotherapy, telemedicine, digital health, patient-reported outcomes, blended care

## Abstract

In the recent past, digital healthcare technologies are experiencing a significant leap in development, with an additional unforeseen acceleration in implementation due to the SARS-CoV-2 pandemic. The increased use of mobile applications as well as communication technologies to search for services and support hold particular advantages for the management of chronic diseases requiring medium- to long-term treatments and regular follow-up visits. Allergen immunotherapy (AIT), requiring regular application of treatment, represents an optimal scenario for feasible digital support. From patient stratification and care pathways, over personalized decision support for complex clinical scenarios, towards a close and flexible patient-doctor communication in blended care settings: the current article summarizes the latest knowledge on the use and potential of digital health technologies in the area of AIT [Fig Figure1].

## Introduction 

Almost 3 years have passed since the SARS-CoV-2 pandemic changed many aspects of everyday life all over the planet. While every citizen experienced the impact of mitigation measures, especially healthcare systems had to adapt and prepare for the continuous provision of health services during every stage of the still ongoing pandemic [1, 2]. Especially in times of necessary contact restrictions, healthcare providers had to implement new strategies to provide the best possible continuous care, also for patients suffering from chronic and non-communicable diseases, such as allergies and asthma [3]. In a very short period, many aspects of outpatient healthcare were implemented remotely, while professional societies and medical associations elaborated guidance documents to support patient triage and the implementation of remote care pathways [4, 5, 6]. In the area of allergy and clinical immunology, published reports and individual examples encouraged the use of digital technologies to support safe remote care [7, 8, 9]. While many healthcare professionals appreciated the new support by digital technologies, and the state of emergency lead to a reimbursement of remote care in several countries, a broad implementation still lies in the future [10, 11]. An example of COVID-19-independent early adoption in the reimbursement system is given by the German Ministry of Health, which regulated the obligatory reimbursement of health apps by statutory health insurances once the app(s) passed a standardized evaluation process [12]. 

In 2021, SARS-CoV-2 vaccination programs advanced and although the pandemic is still ongoing, outpatient care largely moved back to in-person visits with a certain backlog of check-up visits, follow-up care, and medium-term treatments [13]. However, the experiences of the past months do not vanish, and especially positive insights need to be exploited in order to improve everyday patient care in the future [14]. As digitization is taking hold of many spheres of life, also healthcare professionals can benefit from digital support in the implementation of routine activities, especially with regard to repetitive actions and data-based decision support [15, 16]. Particularly medium- to long-term treatments with repeated applications and follow-up visits, such as allergen-specific immunotherapy (AIT) [17] are an excellent application area for digital and/or remote support. This article will summarize different concepts of digital allergology for AIT and point out benefits as well as challenges for their implementation. 

## Data-based treatment prescription 

According to current guidelines and common clinical practice, the decision on AIT prescription is based on the thorough assessment of clinical history and diagnostic in vitro and/or in vivo tests, such as the determination of serum IgE antibodies, skin prick testing, or specific provocation tests [18]. After the diagnostic work-up, clinical significance of the obtained findings needs to be proven before prescribing symptomatic drugs or the to date only causal treatment – AIT. Therapeutic decision-making requires a thorough assessment of symptom severity and duration in combination with an estimation of allergen exposure, as well as information on the intake of symptomatic over-the-counter (OTC) drugs. The evaluation of disease control under symptomatic treatment is particularly useful for the consideration of an AIT [19]. Although the preventive potential of early immunotherapy is widely discussed, particularly patients with mild and intermittent symptoms may benefit sufficiently from symptomatic therapy, and recommendations to date mainly aim at patients for whom this treatment resulted to be insufficient [18]. Overall, the diagnostic procedure and assessment of disease control includes several challenges, which can be tackled with support of digital and mobile health technologies [20]. 

## Digital decision support for an early identification of patients who can benefit from AIT 

Between the first onset of potential allergy symptoms and a proper diagnostic work-up, several years may pass and patients tend to self-medicate with OTC drugs based on assumptions and internet research [21]. As underdiagnosed and undertreated allergic rhinitis not only represents a significant burden with regard to quality of life but also in terms of economic impact, it is desirable to improve the access of patients with symptoms to a proper diagnostic work-up [22]. Possible solutions can be broadly available symptom assessment apps [23, 24], digital diaries indicating a potential correlation between symptoms and exposure [24, 25], as well as clinical decision support systems for primary care doctors and specialists [26, 27]. Examples of validated symptom and exposure monitoring apps are: MASK-Air [19, 20, 25, 28], Hay Fever Diary /Pollen App [29], and AllergyMonitor [24]. While general guidance of users via mobile applications analyzing the clinical history and giving (mostly probabilistic) estimations of a potential pre-diagnosis can guide patients to contact a healthcare professional instead of self-medicating [30], symptom diaries enable the precise and prospective collection of symptoms in preparation for the doctor’s visit [23, 24, 25, 27, 28, 29]. At the primary contact point between patients and healthcare professionals, usually at primary care facilities, general practitioners and pediatricians may be supported by clinical decision support systems (CDSS) in the decision on diagnostic procedures and referral [31, 32]. Finally, pharmacists are an important contact point for patients who do not go to see a doctor. Decision support tools have been proposed to support pharmacists in stratifying patients and recommending adequate OTC medication or refer them to a doctor for diagnosis and potential AIT prescription [33, 34]. 

## Digital support technologies for AIT prescription among polysensitized patients 

In areas with a high prevalence of polysensitization and overlapping pollination periods, the prescription of AIT may be challenging even for specialists. Retrospective clinical histories and multiple positive results in skin prick test (SPT) or IgE to allergen extracts hamper the identification of the eliciting allergen and therefore complicate AIT prescription. For this scenario, a clinical decision support system is under investigation to support the diagnostic work-up of polysensitized patients by taking into account the clinical history, SPT, molecular IgE results, and finally eDiary results matched with local pollen counts. First evaluations among allergists and non-allergists indicate a supportive effect on the diagnostic performance and AIT prescription rate [35]. It is important to note, that none of the previously mentioned technologies aims at substituting a healthcare professional. The common target is to enable informed, joint decision-making and to increase the access to specialized healthcare in a timely manner. 

## Blended care – teaming up personal healthcare with digital support 

In the area of psychotherapy, blended care concepts have been developed and positively evaluated for several diseases, particularly in the area of behavioral therapy for anxiety [36, 37]. Blended care describes the combination of in-person healthcare visits with remote or online training sessions. This enables continuous access to therapy and care without overwhelming the capacities of healthcare systems. In allergy care, blended care concepts have been shown to increase the adherence to mobile symptom monitoring in seasonal allergic rhinitis to more than 80% over several weeks [38]. As the use of monitoring apps drops significantly faster if not accompanied or prescribed by a healthcare professional [25], this may indicate that blended care concepts could be a valuable tool, also for allergy care. Particularly prospective symptom recording in combination with exposure data generates a broad database for clinicians to evaluate the clinical relevance of sensitizations, but also treatment efficacy and possible side effects. 

## eHealth for AIT management 

Once a diagnostic work-up has led to the prescription of AIT, adherence to treatment is an essential aspect regarding efficacy outcomes. While subcutaneous applications of AIT (SCIT) are subjected to a certain control and motivation by healthcare staff due to regular visits for injections, adherence to sublingual immunotherapy (SLIT) depends mostly on the intrinsic motivation and coordination of the patients (or caretakers) themselves [39]. As this challenge is not unique to AIT but holds true for most chronic diseases requiring continuous medication intake, several strategies for digital support have been proposed. From relatively simple reminder systems [40], to more complex models including efficacy feedback [41, 42] and the use of artificial intelligence [43], a broad variety of tools has been developed and partly also evaluated in clinical studies with diverse results [44]. A commonly described challenge for digital support technologies is the fact, that one tool does not fit all patients, but the choice of support should ideally be based on individual needs and preferences. During the SARS-CoV-2 pandemic, video consultations particularly gained importance for patients requiring regular checks and follow-up visits [45]. In allergy care, intermittent remote follow-up visits by video have been described as a feasible tool to assess treatment efficacy, possible side effects and the need for a prescription renewal without the need of travelling for a personal visit [46]. Using telemedicine technologies, such as video consultations and electronic prescription renewals, health care professionals (HCPs) are able to keep close contact to their patients in order to check for individual needs to keep an optimal disease and treatment management. Finally, patient-reported outcomes represent a valuable base for patient stratification and decisions on the continuation or cessation of treatment as efficacy can be evaluated in conjunction with individual exposure levels [20]. Direct feedback of these results in intuitive summaries for patients can also increase intrinsic motivation as success can be directly monitored. 

## Conclusions and future perspectives 

Digital technologies offer a broad spectrum of possibilities to improve the management of patients receiving AIT. Starting with the identification of patients who could benefit from this disease-modifying treatment over data-based, personalized decision support for complex clinical scenarios to close yet flexible patient-doctor communication in blended care settings, the opportunities are manifold. The current COVID-19 pandemic illustrated how efficiently digital technologies support optimal care of patients in this scenario and fostered further innovative development in this field. However, more studies are needed to prove the positive impact of individual technologies on patient stratification, diagnostic work-ups, prescription, adherence, and above all: clinical outcome. 

## Funding 

No funding was received for the production of the present manuscript- 

## Conflict of interest 

Stephanie Dramburg declares personal and speaker’s fees from OMRON Healthcare Co. Ltd., Bencard Allergie GmbH, DBV Technologies, LETI Pharma and Allergopharma. 

**Graphical Abstract Figure1:**